# TGF-β Signaling in Lung Health and Disease

**DOI:** 10.3390/ijms19082460

**Published:** 2018-08-20

**Authors:** Akira Saito, Masafumi Horie, Takahide Nagase

**Affiliations:** 1 Department of Respiratory Medicine, Graduate School of Medicine, The University of Tokyo, 7-3-1 Hongo, Bunkyo-ku, Tokyo 113-0033, Japan; mhorie-tky@umin.ac.jp (M.H.); takahide-tky@umin.ac.jp (T.N.); 2 Division for Health Service Promotion, The University of Tokyo, 7-3-1 Hongo, Bunkyo-ku, Tokyo 113-0033, Japan; 3 Hastings Center for Pulmonary Research, Division of Pulmonary, Critical Care and Sleep Medicine, Department of Medicine, Keck School of Medicine, University of Southern California, Los Angeles, CA 90033, USA

**Keywords:** TGF-β, bronchial asthma, emphysema, pulmonary fibrosis, lung cancer

## Abstract

Transforming growth factor (TGF)-β is an evolutionarily conserved pleiotropic factor that regulates a myriad of biological processes including development, tissue regeneration, immune responses, and tumorigenesis. TGF-β is necessary for lung organogenesis and homeostasis as evidenced by genetically engineered mouse models. TGF-β is crucial for epithelial-mesenchymal interactions during lung branching morphogenesis and alveolarization. Expression and activation of the three TGF-β ligand isoforms in the lungs are temporally and spatially regulated by multiple mechanisms. The lungs are structurally exposed to extrinsic stimuli and pathogens, and are susceptible to inflammation, allergic reactions, and carcinogenesis. Upregulation of TGF-β ligands is observed in major pulmonary diseases, including pulmonary fibrosis, emphysema, bronchial asthma, and lung cancer. TGF-β regulates multiple cellular processes such as growth suppression of epithelial cells, alveolar epithelial cell differentiation, fibroblast activation, and extracellular matrix organization. These effects are closely associated with tissue remodeling in pulmonary fibrosis and emphysema. TGF-β is also central to T cell homeostasis and is deeply involved in asthmatic airway inflammation. TGF-β is the most potent inducer of epithelial-mesenchymal transition in non-small cell lung cancer cells and is pivotal to the development of tumor-promoting microenvironment in the lung cancer tissue. This review summarizes and integrates the current knowledge of TGF-β signaling relevant to lung health and disease.

## 1. Introduction

The transforming growth factor (TGF)-β superfamily consists of three isoforms of TGF-β, Activin, Nodal, bone morphogenetic proteins (BMPs), growth and differentiation factors (GDFs), and others. In mammals, 33 proteins are known to be members of the TGF-β superfamily [1]. TGF-β is the prototypic and most-studied factor, and exhibits versatile functions in almost all cell types. Molecular cloning of human TGF-β was reported in 1985 [2], and its receptors were subsequently identified [3]. Numerous studies over the past decades have reported that TGF-β regulates a wide range of biological processes including cell proliferation, differentiation, apoptosis, extracellular matrix (ECM) synthesis, and stem/progenitor cell fates, thereby affecting embryogenesis, morphogenesis, wound healing, and immune responses of multiple organs. TGF-β family members diversified and adapted to regulate biological systems that evolved over time. TGF-β family members are evolutionarily conserved from arthropods, although TGF-β ligands originated at a later stage of evolution.

Development of the respiratory system is an essential event for vertebrate land adaptation. The fetal lungs are first inflated at birth; thereafter, repeated cycles of respiration continue throughout the entire lifespan. The lungs are structurally exposed to extrinsic stimuli and pathogens, and are susceptible to inflammation, allergic reactions, and carcinogenesis. TGF-β is necessary for lung organogenesis and homeostasis, and is involved in many respiratory diseases, including pulmonary fibrosis, emphysema, bronchial asthma, and lung cancer [4]. In this review, we focus on TGF-β signaling and integrate the current literature to address its role in lung health and disease.

## 2. TGF-β Activation in the Extracellular Milieu

Secretion of TGF-β alone is not sufficient for its bioavailability. TGF-β needs to be activated by several mechanisms, including proteolysis, low pH, reactive oxygen species (ROS), and thrombospondin-1 [5,6]. Specific integrins such as integrin αVβ6 also activate TGF-β by sensing traction forces or stiffness of the ECM [7].

TGF-β peptides are synthesized as latent precursors and are cleaved to form a mature TGF-β dimer noncovalently associated with latency-associated peptide (LAP). LAP of TGF-β1 or TGF-β3 has an integrin recognition motif (RGD sequence) and binds to integrins. The secreted complex of TGF-β and LAP is bound by latent TGF-β binding protein (LTBP), which forms a large latent complex (LLC) [8]. LTBP is a matrix protein incorporated in the ECM, and latent TGF-β (TGF-β-LAP complex) can be stored in the extracellular milieu.

Animal studies have demonstrated functional importance of LTBP isoforms in lung morphogenesis. LTBP-3-null mice display defective lung alveolarization [9]. LTBP-4 is highly expressed in the murine developing lung [10], and is necessary for elastic fiber assembly on microfibrils [11]. LTBP-4-deficient mice show severe pulmonary emphysema [12] due to impaired elastogenesis and structural defects of lung alveoli. These phenotypes are presumably associated with dysregulated TGF-β signaling.

Upon activation, TGF-β is released from the LAP and rapidly mobilized to exert its activity through binding to cell surface receptors. Temporospatial distribution of latent TGF-β and its receptors in the tissue and its dynamic activation contribute to the diversity of TGF-β-mediated biological processes. Intriguingly, a mechanical stress-mediated mechanism of TGF-β activation has been reported [13], and integrins bound to the TGF-β-LAP complex mediate cell contractile forces that lead to TGF-β activation. LTBP incorporated in the ECM is also bound to the LAP that provides mechanical resistance. Such pulling forces result in conformational changes of latent TGF-β and concomitant TGF-β release. Latent TGF-β therefore serves as a sensor for mechanical stress, and released TGF-β acts as an effector. TGF-β itself further regulates expression of integrins and ECM proteins [14,15], which in turn participate in the fine-tuning of TGF-β activation by binding to LAP and LTBP. Because the lung is a highly mechanical organ with a unique alveolar structure, mechanical stress-mediated regulation of TGF-β activity and ECM organization are likely to be important parameters for understanding pulmonary physiology and pathology.

## 3. Context-Dependency of TGF-β Signaling

Activated TGF-β in a dimeric form binds to type I (TGFβR-I, also known as ALK-5) and type II (TGFβR-II) receptors. TGFβR-I is phosphorylated by TGFβR-II upon ligand binding, and in the canonical Smad-dependent pathway, TGFβR-I phosphorylates the intracellular signal transducers, Smad2 and Smad3. Phosphorylated Smad2 and Smad3 interact with Smad4, and translocate into the nucleus [16]. Smad complexes regulate target gene transcription in association with various transcription factors, transcriptional coactivators, and corepressors. In addition to this canonical pathway, TGF-β also activates mitogen-associated protein kinase cascades and RhoA GTPase [17].

TGF-β acts on a wide variety of cell types and induces various cellular responses. For example, TGF-β is involved in stem cell self-renewal and also controls differentiation of multiple cell lineages [18]. Context-dependent responses are regulated by multiple aspects of the TGF-β signaling cascade, including crosstalk with other signals and transcriptional regulation [19]. The three TGF-β ligand isoforms show differential expression patterns and biological effects. Intracellular Smad7 is known to inhibit TGF-β signaling through various mechanisms, and is important as a negative feedback regulator [20,21]. TGF-β receptors and Smad molecules are post-translationally controlled by ubiquitination-mediated degradation [22], and their activity and signaling magnitude are also regulated by protein modifications [23,24].

Cell type specification is regulated by a few master transcription factors, and forced overexpression of such master genes is sufficient for inducing many cell lineages [25]. Recent genome-wide chromatin occupancy and gene expression analyses have revealed that cell type-specific effects of TGF-β are achieved by cooperation between Smad complexes and master transcription factors. For example, induction of Myod1, a master transcription factor for myogenesis, directs Smad3-mediated transactivation of muscle-specific genes [18].

NKX2-1 (also known as thyroid transcription factor-1) is a homeodomain transcription factor expressed in pulmonary epithelial cells. NKX2-1 is a master regulator of lung morphogenesis and is indispensable for tracheoesophageal separation, branching morphogenesis, and alveolar maturation [26]. NKX2-1 is essential for transactivation of lung-specific genes, such as the alveolar epithelial cell-specific surfactant genes (SPA, SPB, and SPC) as well as the club cell-specific gene, CC10 [27]. Convergence of NKX2-1 and TGF-β signaling has been shown to be important for transcriptional regulations in lung epithelial cells. TGF-β represses transcription of SPB, and Smad3 interacts with NKX2-1 and decreases its activity [28]. Moreover, genome-wide chromatin occupancy analysis using lung adenocarcinoma cells has revealed that NKX2-1 colocalizes with Smad3 to regulate a subset of genes while competing with Smad3-Smad4 complex formation resulting in altered genomic binding profiles of Smad3 [29]. Both pro-tumorigenic and tumor-suppressive roles of NKX2-1 have been reported in lung adenocarcinoma [30]. NKX2-1 and TGF-β signaling therefore appear to be a key component for regulating lung adenocarcinoma cell features.

## 4. TGF-β Signaling in Lung Organogenesis

Lung development takes place through coordinated growth and differentiation of the endoderm-derived epithelium and the mesoderm-derived mesenchyme. During the embryonic stage, lung buds arise from the anterior foregut, and branching morphogenesis follows during the pseudoglandular stage [31].

Lung morphogenesis is controlled by multiple signals such as fibroblast growth factor, sonic hedgehog (SHH), Wnt/β-catenin, and BMP. In cooperation with these signals, TGF-β plays key roles in epithelial-mesenchymal interactions. During the pseudoglandular stage, the three isoforms of TGF-β show different expression patterns [32]. TGF-β1 is expressed throughout the mesenchyme and is highly localized in the area underlying the epithelial branching point. TGF-β2 is localized in the distal epithelium, and TGF-β3 is detected in the proximal mesenchyme and distal epithelium.

Different pulmonary phenotypes have been reported in the knockout mice of TGF-β isoforms, indicating distinct functions for the different isoforms (Table 1). TGF-β1-deficient mice show systemic inflammation, and in the lungs, generalized perivasculitis or interstitial pneumonia is observed [33,34]. TGF-β2-null mice have postnatal lung defects with collapsed distal airways [35]. TGF-β3 deficiency leads to defective lungs with alveolar hypoplasia and mesenchymal thickening [36].

Conditional abrogation of TGFβR-II and TGF-β signaling inhibition in SPC-expressing lung epithelial cells result in retarded postnatal alveologenesis, but without an apparent prenatal phenotype [37]. Conditional TGFβR-II knockout mice generated using *Nkx2-1-Cre* display abnormal alveolarization and emphysema [38]. Smad3-deficient mice show impaired alveolarization and centrilobular emphysema [39,40], similar to the effect of TGFβR-II abrogation in lung epithelial cells. Notably, deletion of TGFβR-I in epithelial cells using *Gata5-Cre* leads to immature alveoli and a disorganized epithelium with reduced club cell population [41]. All of these indicate that TGF-β signaling is necessary for lung epithelial cell differentiation and maturation.

Mesenchymal abrogation of TGFβR-II disrupts lung branching morphogenesis, resulting in cystic malformation of the bronchi. This phenotype was shown to be associated with dysregulated SHH signaling in the mesenchyme [42]. Mesodermal inactivation of TGFβR-I results in pulmonary hypoplasia due to impaired differentiation of mesodermal progenitor cells [43].

Ectopic expression of TGF-β1 in the lung epithelium disrupts lung morphogenesis and perturbs epithelial differentiation [44]. Moreover, exogenous TGF-β exerts an inhibitory effect on lung branching morphogenesis as demonstrated in explant cultures [45]. 

**Figure 1:**
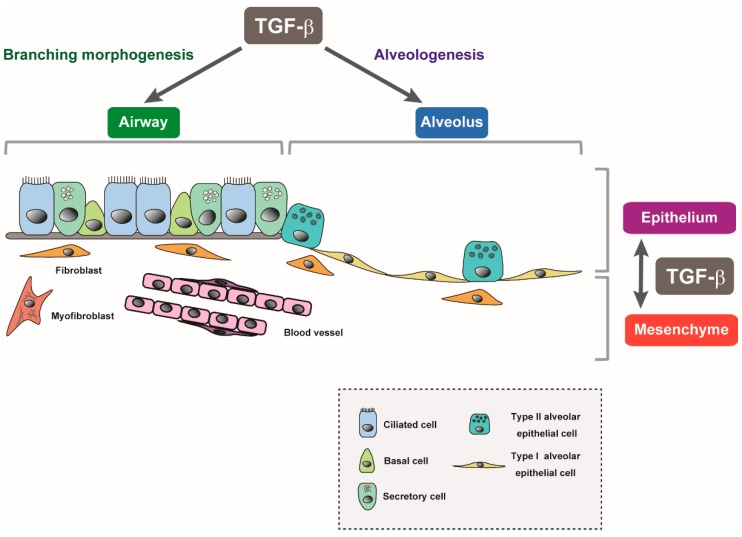
Structure of the airway and alveolus. TGF-β regulates epithelial-mesenchymal interactions and is crucial for branching morphogenesis and alveologenesis during development.

Taken together, TGF-β signaling appears to play distinct and critical roles in the lung epithelium and mesenchyme, and is required for epithelial-mesenchymal interactions to achieve lung branching morphogenesis and alveologenesis (Figure 1).

## 5. TGF-β Signaling in Lung Alveolar Epithelial Growth and Differentiation

Differentiated airway epithelial cells include basal, secretory, ciliated, and neuroendocrine cells, and the alveoli are lined by alveolar epithelial type I and type II cells (Figure 1). Alveolar epithelial type I cells cover the majority of the alveolar surface, allowing for gas exchange, while type II cells are involved in pulmonary surfactant production [46]. As previously mentioned, analyses of genetically engineered mouse models have revealed crucial roles for TGF-β signaling in lung epithelial growth and differentiation (Table 1).

**Figure 2:**
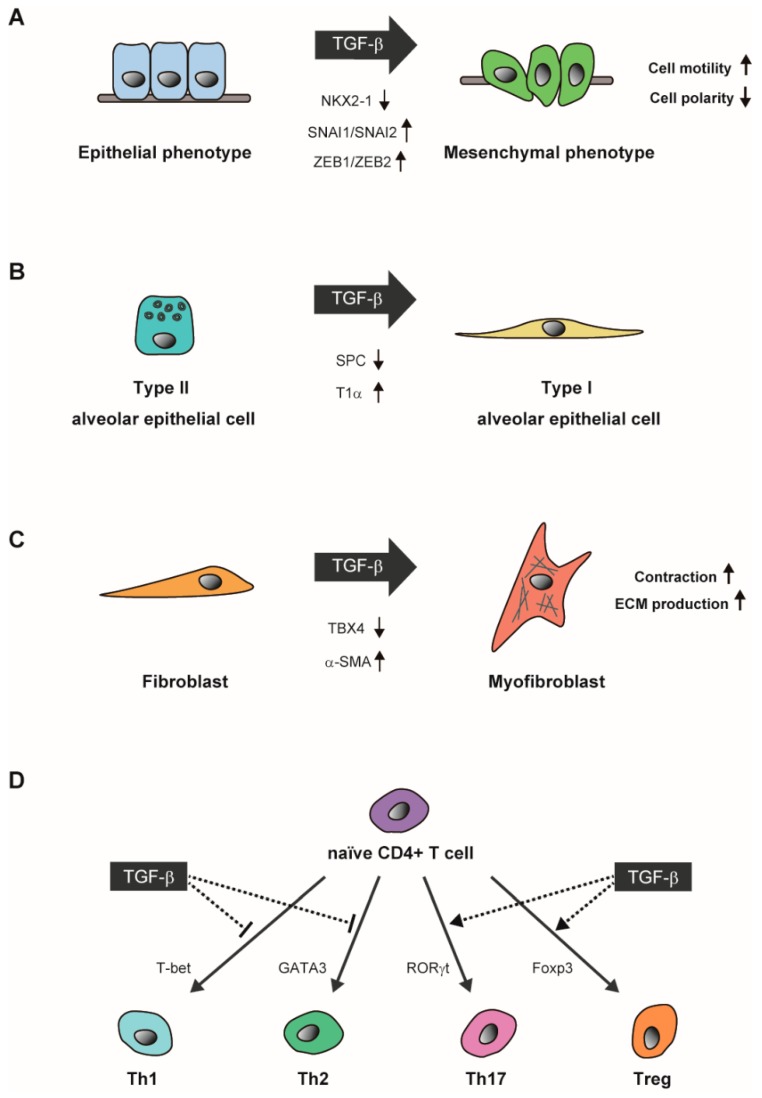
Context-dependent action of TGF-β. (**A**) TGF-β promotes epithelial-mesenchymal transition (EMT) in alveolar epithelial cells to confer a mesenchymal phenotype, or in lung cancer cells to enhance migratory and invasive capacities. TGF-β induces EMT-related transcriptional repressors (SNAI1/SNAI2 and ZEB1/ZEB2) and inhibits the action of NKX2-1, a homeodomain transcription factor important for lung epithelial cell differentiation; (**B**) TGF-β promotes transdifferentiation of surfactant protein C (SPC)-positive type II alveolar epithelial cells to type I alveolar epithelial cells that express podoplanin (T1α); (**C**) TGF-β promotes transdifferentiation of lung fibroblasts to myofibroblasts positive for α-smooth muscle actin (α-SMA), and downregulates TBX4, a T-box family transcription factor unique to lung fibroblasts; (**D**) TGF-β suppresses induction of T helper type 1 (Th1) and type 2 (Th2) cells while positively regulating cell lineage specification from naïve CD^4+^ T cells to Th17 and regulatory T (Treg) cells. T-bet, GATA3, RORγt, and Foxp3 are master transcription factors for Th1, Th2, Th17, and Treg cells, respectively.

It is widely accepted that TGF-β shows cytostatic effects in most epithelial cells, and TGF-β has been shown to inhibit proliferation of alveolar epithelial type II cells [47]. TGF-β is also known as the most powerful inducer of epithelial-mesenchymal transition (EMT) [48]. EMT is a biological process where polarized epithelial cells acquire mesenchymal phenotypes with enhanced cell motility. Mechanistically, TGF-β induces transcriptional repressors, SNAI1, SNAI2, ZEB1, and ZEB2, which subsequently repress adherens junction and tight junction proteins such as E-cadherin and ZO-1, thereby disrupting epithelial cell junction and apical-basal polarity [49] (Figure 2A). ZEB1 and ZEB2 also repress the miRNA-200 family and miR-205, which target ZEB1 and ZEB2, and thereby derepress the EMT regulatory axis of TGF-β and ZEB proteins [50]. TGF-β induces EMT in rat alveolar epithelial cells, which may contribute to disease conditions by endowing epithelial cells with migratory and anti-apoptotic phenotypes [51,52].

A series of pioneering studies established primary cultures of alveolar epithelial type II cells that form polarized monolayers and spontaneously differentiate into type I cells [53]. TGF-β inhibits expression of alveolar epithelial type II cell markers and enhances differentiation of cultured type II cells into type I cells [54] (Figure 2B). Thus, the effects of TGF-β in alveolar epithelial type II cells include EMT induction and type I cell differentiation, which may depend on cell culture conditions.

It is thought that alveolar epithelial type II cells contribute to alveolar homeostasis by self-renewing and differentiating into type I cells [55]. Conversely, a subset of alveolar epithelial type I cells generate type II cells, illustrating the plasticity of alveolar epithelial cells [56]. Importantly, conversion of type I to type II cells is facilitated by TGF-β signaling inhibition in organoid culture [56]. 

Recently, cell culture methods to induce alveolar epithelial cells from pluripotent stem cells have been developed [57,58]. Inhibition of TGF-β signaling has been shown to augment in vitro differentiation of anterior foregut endoderm cells that give rise to NKX2-1-positive alveolar epithelial progenitors [59].

Taken together, TGF-β plays multifaceted roles in alveolar epithelial cells, including cell growth suppression, EMT, and regulating reciprocal differentiation between type I and type II cells.

## 6. TGF-β Signaling in Pulmonary Diseases

### 6.1. TGF-β Signaling in Pulmonary Fibrosis and Emphysema

Pulmonary fibrosis is a chronic and progressive lung disease, in which repeated wound and repair processes lead to irreversible structural alterations and tissue stiffening [60]. Pathophysiological steps include alveolar epithelial damage by extrinsic irritants, fibroblast activation, and persistent fibrotic reaction. Differentiation of lung fibroblasts into myofibroblasts is a key step in the development of tissue fibrosis. Myofibroblasts express α-smooth muscle actin (α-SMA) as a marker of activated fibroblasts, and are capable of ECM production including collagen, laminin, and fibronectin. TGF-β is the most potent factor for the induction of myofibroblast differentiation (Figure 2C), and increased expression of TGF-β has been reported in fibrotic lungs. The major cellular sources of TGF-β in pulmonary fibrosis have been shown to be alveolar macrophages and metaplastic type II alveolar epithelial cells [61,62]. In addition to ECM components, TGF-β induces integrins, matrix metalloproteinases, protease inhibitors, and regulators of small GTPases [14,15]. These molecules participate in tissue remodeling and influence cell-ECM interactions. In addition, TGF-β is thought to promote lung fibrosis by suppressing production of anti-fibrotic molecules such as hepatocyte growth factor and prostaglandin E2 [63,64]. Furthermore, TGF-β inhibits alveolar epithelial cell growth and repair. Thus, TGF-β is a key player in fibrotic processes, acting on both fibroblasts and alveolar epithelial cells.

Smoking-induced emphysema is the main cause of chronic obstructive pulmonary disease (COPD), and is characterized by lung hyperinflation and enlargement of air spaces distal to the terminal bronchioles. Recently, an association between *TGFB2* polymorphisms and COPD has been reported [65]. TGF-β1 expression has been shown in small airway epithelial cells among smokers and patients with COPD [66], suggesting that pathologically activated TGF-β signaling is involved in the pathogenesis of emphysema.

In murine models, fibrosis and emphysema are often discussed as contrasting disorders. As previously mentioned, conditional deletion of TGFβR-II in lung epithelial cells results in emphysema-like phenotypes whereas these mice are protected from bleomycin-induced pulmonary fibrosis [37,38]. Smad3-deficient mice show progressive alveolar destruction resembling emphysema while they are protected from bleomycin-induced lung fibrosis [67]. Itgb6-null mice develop age-related emphysema [68], similar to the effects of Smad3 deficiency [39,40] or TGFβR-II abrogation in lung epithelial cells [37,38]. Supporting the notion that the phenotype of Itgb6 deletion is caused by disruption of integrin αvβ6-mediated TGF-β activation, ectopic expression of active TGF-β1 overcomes the effect of Itgb6 deletion [68]. On the other hand, Itgb6-null mice are resistant to bleomycin-induced lung injury and fibrosis [69]. Another study consistently showed that αvβ6-blocking antibody attenuated bleomycin-induced pulmonary fibrosis [70]. These models suggest that lack of TGF-β signaling predisposes the lungs to develop emphysema while conferring resistance to fibrosis.

**Figure 3:**
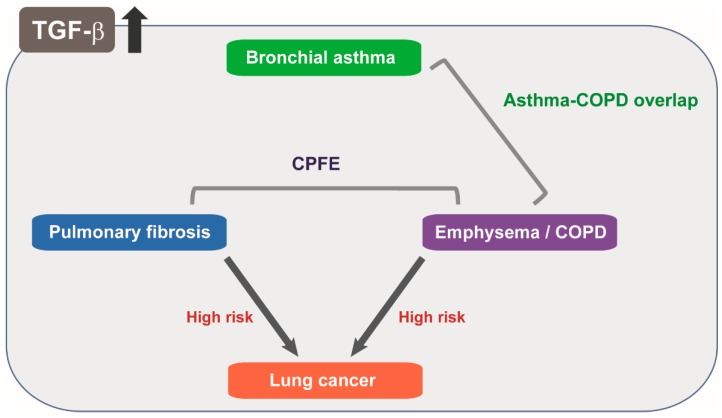
Upregulation of TGF-β ligands is observed in bronchial asthma, pulmonary fibrosis, emphysema, and lung cancer. Combined pulmonary fibrosis and emphysema (CPFE) and asthma-COPD overlap are increasingly recognized as distinct entities. Pulmonary fibrosis and emphysema are known as independent risk factors for lung cancer.

In contrast to this simplified dichotomy in mouse models, the coexistence of fibrosis and emphysema in the same patient is common. Of clinical importance, pulmonary fibrosis and COPD are independent risk factors for lung cancer (Figure 3). Notably, combined pulmonary fibrosis and emphysema (CPFE) is increasingly recognized as an entity with severely impaired gas exchange and higher frequencies of pulmonary hypertension and lung cancer [71] (Figure 3). Pathologically activated TGF-β signaling in relation to smoking and ageing are involved in emphysema and fibrosis, and common molecular mechanisms have therefore been postulated in both diseases. Because dynamic organization and turnover of the ECM in the lung tissue is largely regulated by TGF-β, these processes might represent different forms of tissue remodeling as a consequence of pathologically activated TGF-β signaling. However, it remains largely unknown how TGF-β signaling differentially contributes to these two contrasting phenotypes.

### 6.2. Dual Roles of TGF-β in Asthma Involving Immune Response and Airway Remodeling

Bronchial asthma is a global health problem that affects more than 300 million people worldwide [72]. Asthma is characterized by chronic airway inflammation and hyperresponsiveness mediated by T helper type 2 (Th2) cells and the related cytokines, interleukin (IL)-4, IL-5, IL-9, and IL-13. These cytokines cause chronic inflammation, pulmonary eosinophilia, mucus cell hyperplasia, smooth muscle contraction, and airway remodeling. In addition to Th2 cells, Th17 cells that secrete IL-17A and IL-17F also participate in the development of allergic airway inflammation [72]. Inhaled corticosteroids have greatly improved the management of asthma in recent decades. However, some asthmatic patients are refractory to this therapy because of irreversible narrowing of the airway [73]. Thickening of the airway smooth muscle layer, a hallmark of asthmatic airway remodeling, is related to the clinical severity of asthma [74]. Recently, increasing attention has been focused on asthma and COPD overlap as a clinical entity (Figure 3).

The importance of TGF-β signaling in the pathogenesis of asthma has been illustrated by genome-wide association studies. It has been reported that *SMAD3* polymorphism is associated with asthma [75]. A meta-analysis further suggested that C509T or T869C polymorphism of *TGFB1* gene may predispose to asthma [76]. Moreover, a recent study reported that patients with Loeys-Dietz syndrome who harbor loss-of-function mutations in TGFβR-I or TGFβR-II frequently develop allergic diseases including asthma [77].

TGF-β1 concentration in bronchoalveolar lavage fluid is elevated in atopic asthma [78], and TGF-β expression is increased in bronchial specimens of asthmatic patients [79]. Bronchial epithelial cells and eosinophils are the major source of TGF-β in the asthmatic airway [80]. TGF-β enhances airway smooth muscle proliferation and ECM deposition by activated fibroblasts, eventually leading to structural alterations of the airway. Moreover, it has been reported that TGF-β1 modulates airway smooth muscle shortening and hyperresponsiveness by enhancing excitation-contraction coupling [81].

The pathological role of TGF-β in asthma is not restricted to airway remodeling, and its effect on the immune response is thought to be more important than previously recognized. TGF-β induces Foxp3 gene expression in CD4+ CD25- naïve T cells in the presence of T cell receptor stimulation, which in turn mediates differentiation of regulatory T (Treg) cells with immunosuppressive functions [82] (Figure 2D). Notably, TGF-β is further produced by Treg cells. TGF-β1-null mice consistently fail to maintain peripheral Treg cells, resembling the phenotype of Foxp3-null mice [83]. Of therapeutic importance, it has been reported that adoptive transfer of Treg cells ameliorates allergic airway inflammation in a murine model [84]. T cell-specific deletion of TGFβR-II results in a reduction of peripheral Treg cells, and also inhibits thymic CD8+ T cell maturation and natural killer T (NKT) cell development in the thymus [85]. CD4+ T helper cells differentiate into Th1, Th2, and Th17 cells characterized by the signature cytokines, IFN-γ, IL-4, and IL-17, respectively [86]. TGF-β is known to induce Th17 cells in combination with IL-6, which contribute to neutrophilic recruitment into the airway and Th2 cell-mediated eosinophilic inflammation [87,88]. In contrast, TGF-β has inhibitory effects on Th1 and Th2 cell differentiation through suppression of T-bet and GATA3 transcription factors, respectively [89] (Figure 2D). Thus, TGF-β plays multimodal roles in Th2 type immune response. TGF-β suppresses Th2 cells directly or through induction of Treg cells. On the other hand, TGF-β also enhances Th2 cell-mediated action by inducing Th17 cells.

Collectively, TGF-β positively regulates Treg, Th17, NKT, and CD8+ T cells while inhibiting Th1 and Th2 differentiation. Thus, TGF-β plays multifunctional roles in T cell differentiation and homeostasis, thereby affecting the immune system in the asthmatic airway. 

### 6.3. TGF-β Mediates EMT in Non-Small Cell Lung Cancer (NSCLC)

Lung cancer is the leading cause of cancer-related mortality worldwide [90]. NSCLC comprises the majority of lung cancers, which include the histological subtypes of adenocarcinoma and squamous cell carcinoma. Higher TGF-β expression levels are associated with lymph node metastasis and tumor angiogenesis in NSCLC [91], and tumor cells established from NSCLC express TGF-β ligands.

It is widely believed that TGF-β plays dual roles during tumor progression [92]. TGF-β suppresses epithelial cell proliferation and acts as a tumor suppressor in the early stage of tumorigenesis, and loss-of-function mutations in TGF-β signaling components have been identified in several cancer types. Inactivating mutations of TGFβR-II are frequent in colorectal carcinomas with microsatellite instability [93], and loss of chromosomal region 18q21, encoding *SMAD4* gene, is found in pancreatic and colorectal cancers [94]. Mutations of TGFβR-II and SMAD4 are rare in NSCLC [95], whereas epidermal growth factor receptor (EGFR) and KRAS mutations are frequently found in lung adenocarcinomas [96].

In addition to its cytostatic effects, TGF-β potently induces EMT in cancer cells, which represents a tumor-promoting aspect of TGF-β activity in the later stages of cancer progression. Cultured cancer cells usually lack cell polarity or epithelial integrity, and remain in an intermediary state of partial or incomplete EMT [97]. NSCLC cell lines could therefore be classified into epithelial-like and mesenchymal-like subgroups based on gene expression profiling [98,99]. Recent studies using NSCLC cell lines have focused on the TGF-β-mediated completion of EMT programs.

TGF-β-mediated EMT is modified by crosstalk with other signaling pathways. In A549 lung adenocarcinoma cells, TNF-α enhances EMT and induces cancer cells with a secretory phenotype capable of producing various cytokines and chemokines [14]. Of particular importance is the convergence between oncogenic KRAS and TGF-β signaling in EMT processes [100]. Transduction of oncogenic KRAS dramatically enhances TGF-β-elicited SNAI1 expression, and TGF-β induces SNAI1 more strongly in cancer cells harboring constitutively active KRAS mutations.

TGF-β-mediated EMT in cancer cells is associated with aggressive features such as resistance to apoptosis, acquisition of stem cell traits, and chemoresistance [101]. On the other hand, recent studies have suggested that EMT is likely dispensable for metastasis [102].

### 6.4. TGF-β Orchestrates the Tumor Microenvironment

Besides its direct effect on cancer cells, TGF-β facilitates invasion and metastatic spread through reciprocal interactions between cancer cells and the tumor stromal microenvironment [103]. TGF-β orchestrates the development of tumor stroma and promotes angiogenesis, immune evasion, and remodeling of the ECM [104,105]. The stromal reaction presumably mediated by TGF-β is associated with poor prognosis in resected lung adenocarcinomas [106]. Tumor stroma comprises cancer-associated fibroblasts (CAFs), immune cells, blood vessels, and the ECM [107]. CAFs are activated by TGF-β and promote tumor progression by secretion of soluble factors and ECM remodeling [108,109]. The gene signatures related to TGF-β signaling are enriched in CAFs isolated from NSCLC tissues compared to their normal counterparts [110]. Tumor stroma shows dysregulated ECM organization and tissue stiffness, which are similar to the features of pulmonary fibrosis. Of clinical importance, cancer tissue fibrosis is associated with increased interstitial fluid pressure, which impedes delivery of cancer therapeutics [111].

Tumor angiogenesis is crucial for the delivery of oxygen and nutrients to cancer cells. TGF-β is known to stimulate angiogenic factors such as vascular endothelial growth factor and connective tissue growth factor produced by cancer cells or stromal fibroblasts. On the other hand, it has been suggested that TGF-β negatively regulates lymphangiogenesis [112].

Cancer progression is thought to be dependent on immunosurveillance evasion, and this notion is supported by the recent success of immune checkpoint inhibitors as therapeutic options for NSCLC. As previously mentioned, TGF-β participates in the maintenance of T cell homeostasis and induces Treg cells that limit tumor immune responses [82]. Moreover, TGF-β in the cancer tissue polarizes tumor-associated macrophages, myeloid-derived suppressor cells, and tumor-associated neutrophils to tumor-promoting phenotypes [113,114]. It has also been reported that TGF-β signaling blockade by TGF-β blocking antibody or TGFβR-I kinase inhibitor enhances anti-tumor immunity and shows therapeutic benefits [115,116].

Taken together, TGF-β facilitates cancer progression through diverse host-tumor interactions, including fibroblast activation, ECM remodeling, angiogenesis, and immune evasion.

### 6.5. TGF-β Signaling in Small Cell Lung Cancer (SCLC)

SCLC is a highly aggressive lung cancer subtype that accounts for 10–15% of lung cancers [117]. SCLC originates from lung neuroendocrine cell precursors, and achaete-scute complex homolog 1 (ASCL1) is a master transcription factor for neuroendocrine differentiation [118]. SCLC cell lines and tissue samples contain heterogeneous gene expression patterns. A variant subtype of SCLC with poor neuroendocrine differentiation and low ASCL1 expression has been recently reported [119,120].

Most classic SCLC cell lines lack TGFβR-II expression and are unresponsive to TGF-β [121], whereas variant SCLC cell lines tend to express relatively high levels of TGFβR-II. A recent report described molecular mechanisms responsible for this SCLC heterogeneity [122]. In lung epithelial/cancer cells with TGFβR-II expression, endogenous TGF-β suppresses ASCL1 expression. However, TGF-β signaling is inactivated in classic SCLC cells and ASCL1 expression is derepressed. Because TGF-β signaling inactivation and ASCL1 induction can promote SCLC survival, loss of TGF-β responsiveness is probably an important aspect of SCLC pathogenesis. Detailed analyses of genomic profiles in heterogeneous SCLC tumor samples may be helpful to dissect the exact role of TGF-β signaling in SCLC and its relation to neuroendocrine differentiation.

## 7. Gene Regulatory Networks Responsible for the TGF-β-Induced Cellular Response

The genome, epigenome, and transcriptome in a broad range of cell types and disease conditions have been characterized using recent advances in next-generation sequencing technology. Histone modification, DNA methylation, and gene expression profiling data across the whole genome have been included in public databases [123]. Genome-wide chromatin-binding profiling data have further provided mechanistic insights into transcriptional regulations [124]. It is becoming clear that a subset of transcription factors control cell lineage determination through activation of cell type-specific super-enhancers, which are enriched with active histone marks and defined as large genomic regions occupied by high densities of master transcription factors [125,126]. In parallel with these advances in genome biology, context-dependent transcriptional regulations by TGF-β-Smad pathway is being elucidated in more detail.

It has been reported that T-box family genes such as TBX4 are associated with super-enhancers and are involved in defining cellular identity of lung fibroblasts. TGF-β potently suppresses T-box family genes while inducing myofibroblast differentiation, and super-enhancers unique to lung fibroblasts are globally suppressed in lung CAFs. Thus, it has been proposed that TGF-β induces redistribution of super-enhancers at the expense of cell type-specific basal super-enhancers [127].

In the process of TGF-β-mediated EMT in A549 lung adenocarcinoma cells, a few transcription factors associated with super-enhancers (HNF4A, JUNB, and ETS2) have been shown to constitute a gene regulatory network that synergistically induces EMT [128]. As such, TGF-β-mediated cell differentiation or phenotypic conversion is coupled with dynamic reorganization of core transcriptional networks that involve the Smad complex, master transcription factors, and super-enhancers.

## 8. Future Perspectives

TGF-β is essential for lung organogenesis, homeostasis, and pathological conditions. Because TGF-β is involved in the pathogenesis of pulmonary fibrosis and NSCLC, there have been clinical trials testing the efficacy of inhibitors, antisense oligonucleotides, and neutralizing antibodies targeting TGF-β [129]. Moreover, TGF-β inhibition is emerging as a promising strategy for augmenting cancer immunotherapy [115,116].

Recently, single cell transcriptome analysis has become a powerful tool for investigating heterogeneous cell populations and differentiation processes in various conditions [130,131]. In-depth analyses using single cell-based technologies could further elucidate molecular mechanisms underlying TGF-β-mediated cell type-specific responses in physiological and pathological processes.

## Figures and Tables

**Table 1:** Genetically engineered mouse models that demonstrate the roles of TGF-β signaling in lung organogenesis.

Mouse Model	Induction	Target Cell	Phenotype in the Lungs	Phenotype in Other Organs	Reference
*Tgfb1* (^−/−^)			perivasculitis with lymphocytic and plasmacytic infiltration	systemic inflammation	[33]
*Tgfb1* (^−/−^)			perivasculitis with lymphocytic and plasmacytic infiltration; interstitial pneumonia	systemic inflammation	[34]
*Tgfb2* (^−/−^)			collapsed distal airways with dilated conducting airways	Cardiac, craniofacial, limb, spinal column, eye, inner ear, and urogenital defects	[35]
*Tgfb3* (^−/−^)			atelectatic, pseudograndular histology with alveolar hypoplasia; mesenchymal thickening; extensive intrapulmonary and pleural hemorrhage; dilated conducting airways	cleft palate	[36]
*Smad3* (^−/−^)			progressive lung airspace enlargement and emphysematous changes	defects in immune function with inflammatory lesions (originally reported by Yang X et al. in 1999)	[39]
*Smad3* (^−/−^)			reduced pulmonary alveolarization and subsequent centrilobular emphysema	decreased growth rate (originally reported by Datto MB et al. in 1999)	[40]
*T* *gfbr2* ^flox/flox^	crossed with *SPC-rtTA*/*TetO-Cre* mice	lung epithelial cells	retardation of postnatal lung alveolarization with markedly decreased type I alveolar epithelial cells		[37]
*T* *gfbr2* ^flox/flox^	crossed with *Dermo1-Cre* mice	mesoderm-derived tissue including lung mesenchyme	abnormal lung branching and reduced cell proliferation	defective secondary ventral body wall formation, congenital diaphragmatic hernia, and abnormal cardiac development	[37]
*T* *gfbr2* ^flox/flox^	crossed with *Dermo1-Cre* mice	mesoderm-derived tissue including lung mesenchyme	failure in branching morphogenesis and cystic airway malformations	abnormalities in multiple organs	[42]
*T* *gfbr2* ^flox/flox^	crossed with *Nkx2-1-Cre* mice	lung epithelial cells	alveolar enlargement and non-progressive emphysema; resistance to TGF-β-mediated, bleomycin-induced lung injury		[38]
*T* *gfbr1* ^flox/flox^	crossed with *GATA5-Cre* mice	embryonic lung epithelium	immature alveoli and formation of a disorganized and multi-layered epithelium in the proximal airways; marked reduction in the number of club cells		[41]
*T* *gfbr1* ^flox/flox^	crossed with *Dermo1-Cre* mice	mesoderm-derived tissue including lung mesenchyme	reduced submesothelial mesenchyme; restricted α-SMA-positive cell fate and promoted lipofibroblast differentiation; defective epithelial differentiation; disrupted pulmonary vasculogenesis		[43]
